# Supplementary material for: Reconciling Mining with the Conservation of Cave Biodiversity: A Quantitative Baseline to Help Establish Conservation Priorities
Source: PLoS One. 2016 Dec 20;11(12):e0168348. doi: 10.1371/journal.pone.0168348 (PMC5173368; doi:10.1371/journal.pone.0168348)
Supplement: S1 Dataset — (ZIP) [file pone.0168348.s002.zip › Taxa/Serra Norte/SN_2013/N4WS/Lista N4WS-05.pdf]

CAVIDADE N4WS-0005

Classe	Ordem	Fam/Outros	Gên/Outros	Espécie	S-U	U-S	Zon.
Annelida	Oligochaeta			sp.		1	E
Arachnida	Acari			jovem		X	E
Arachnida	Amblypygi	Phrynidae	*Heterophrynus*	*longicornis*	1		E
Arachnida	Araneae	Ctenidae		jovem	2		E
Arachnida	Araneae	Pholcidae	*Mesabolivar*	*aurantiacus*	X		E
Arachnida	Araneae	Pholcidae	*Mesabolivar*	*eberhard*	X		E
Arachnida	Araneae	Symphytognathidae	*Anapistula*	sp.1		X	E
Arachnida	Araneae	Theraphosidae	aff. *Holothele*	sp.1	2		E
Arachnida	Araneae	Theridiidae	*Theridion*	sp.2		X	E
Arachnida	Araneae	Theridiosomatidae	*Plato*	sp.1	X	X	E
Arachnida	Pseudoscorpiones	Chernetidae	*Spelaeochernes*	sp.1		X	E
Entognatha	Collembola	Isotomidae		sp.1		X	E
Insecta	Coleoptera	Tenebrionidae		jovem		X	E
Insecta	Diptera	Psychodidae	*Sciopemyia*	*sordellii*	X	X	E
Insecta	Diptera	Tipulidae	Tipulinae	sp.	X		E
Insecta	Heteroptera	Reduviidae	Emesinae	jovem		1	E
Insecta	Heteroptera	Reduviidae	Reduviinae	jovem		6	E
Insecta	Homoptera	Cixiidae		jovem		X	E
Insecta	Hymenoptera	Chalcidoidea		sp.1		X	E
Insecta	Hymenoptera	Formicidae	*Pheidole*	sp.2		X	E
Insecta	Lepidoptera	Noctuoidea		sp.1	1		E
Insecta	Lepidoptera	Noctuoidea		sp.2		X	E
Insecta	Neuroptera	Myrmeleontidae		jovem		1	E
Insecta	Orthoptera	Phalangopsidae	*Paraclodes*	sp.	5	2	E
Insecta	Orthoptera	Phalangopsidae	*Phalangopsis*	sp.	2		E
Insecta	Psocoptera	Psocomorpha		jovem	X	X	E
Gastropoda	Pulmonata	Systrophiidae	*Happia*	sp.		X	E
Malacostraca	Isopoda	Armadillidae		sp.1		X	E
Malacostraca	Isopoda	Dubioniscidae		sp.1		X	E
Malacostraca	Isopoda	Philosciidae		sp.1		X	E
Amphibia	Anura	Strabomantidae	*Pristimantis*	cf. *fenestratus*	1		E
